# Chimeric Antigen Receptor T-Cell Postinfusion Fever: Infection Profile, Clinical Parameters, and Biomarkers Trends to Assist Antibiotic Stewardship

**DOI:** 10.1093/ofid/ofae398

**Published:** 2024-07-11

**Authors:** Olivier Peyrony, Nicole Garcia-Pouton, Mariana Chumbita, Christian Teijon-Lumbreras, Tommaso Francesco Aiello, Patricia Monzó-Gallo, Antonio Gallardo-Pizarro, Valentín Ortiz-Maldonado, Núria Martinez-Cibrian, Julio Delgado, Carlos Fernandez de Larrea, Josep Mensa, Pedro Puerta-Alcalde, Alex Soriano, Carolina Garcia-Vidal

**Affiliations:** Department of Infectious Diseases, Hospital Clinic of Barcelona, Barcelona, Spain; Emergency Department, Hôpital Saint-Louis, Assistance Publique–Hôpitaux de Paris, Paris, France; Department of Infectious Diseases, Hospital Clinic of Barcelona, Instituto de Investigaciones Biomédicas August Pi i Sunyer, University of Barcelona, Barcelona, Spain; Department of Infectious Diseases, Hospital Clinic of Barcelona, Instituto de Investigaciones Biomédicas August Pi i Sunyer, University of Barcelona, Barcelona, Spain; Department of Infectious Diseases, Hospital Clinic of Barcelona, Instituto de Investigaciones Biomédicas August Pi i Sunyer, University of Barcelona, Barcelona, Spain; Department of Infectious Diseases, Hospital Clinic of Barcelona, Instituto de Investigaciones Biomédicas August Pi i Sunyer, University of Barcelona, Barcelona, Spain; Department of Infectious Diseases, Hospital Clinic of Barcelona, Instituto de Investigaciones Biomédicas August Pi i Sunyer, University of Barcelona, Barcelona, Spain; Department of Infectious Diseases, Hospital Clinic of Barcelona, Instituto de Investigaciones Biomédicas August Pi i Sunyer, University of Barcelona, Barcelona, Spain; Department of Hematology, Hospital Clinic of Barcelona, Instituto de Investigaciones Biomédicas August Pi i Sunyer, University of Barcelona, Barcelona, Spain; Department of Hematology, Hospital Clinic of Barcelona, Instituto de Investigaciones Biomédicas August Pi i Sunyer, University of Barcelona, Barcelona, Spain; Department of Hematology, Hospital Clinic of Barcelona, Instituto de Investigaciones Biomédicas August Pi i Sunyer, University of Barcelona, Barcelona, Spain; Department of Hematology, Hospital Clinic of Barcelona, Instituto de Investigaciones Biomédicas August Pi i Sunyer, University of Barcelona, Barcelona, Spain; Department of Infectious Diseases, Hospital Clinic of Barcelona, Instituto de Investigaciones Biomédicas August Pi i Sunyer, University of Barcelona, Barcelona, Spain; Department of Infectious Diseases, Hospital Clinic of Barcelona, Instituto de Investigaciones Biomédicas August Pi i Sunyer, University of Barcelona, Barcelona, Spain; Department of Infectious Diseases, Hospital Clinic of Barcelona, Instituto de Investigaciones Biomédicas August Pi i Sunyer, University of Barcelona, Barcelona, Spain; CIBERINFECT, Centro de Investigación Biomédica en Red, Instituto de Salud Carlos III, Madrid, Spain; Department of Infectious Diseases, Hospital Clinic of Barcelona, Instituto de Investigaciones Biomédicas August Pi i Sunyer, University of Barcelona, Barcelona, Spain

**Keywords:** CAR T cell, fever, hematology, immunotherapy, infection

## Abstract

**Background:**

This study aimed to describe documented infections associated with postinfusion fever after CAR T-cell therapy and to evaluate daily changes in vital signs, laboratory results, and the National Early Warning Score (NEWS) in patients with and without confirmed bacterial infections following fever onset, with the objective of assisting in antibiotic stewardship.

**Methods:**

This was a retrospective, observational study including all consecutive adult patients who received CAR T-cell therapy. Documented infection in the first fever episode after infusion, and clinical and analytic trend comparison of patients with bacterial documented infections and those without documented infections, are described.

**Results:**

Among 152 patients treated with CAR T-cell therapy, 87 (57.2%) had fever within 30 days of infusion, with a median time from infusion to fever of 3 (interquartile range, 2–5) days. Of these 87 patients, 82 (94.3%) received broad-spectrum antibiotics. Infection was documented in 9 (10.3%) patients and only 4 (4.6%) had bacterial infections. Clinical signs and biomarkers were similar in patients with bacterial documented infection and in those without documented infection at fever onset. Fever, tachycardia, and high C-reactive protein levels remained high during the first 3 days after CAR T-cell infusion, even when no infection was documented.

**Conclusions:**

Fever is a common symptom following CAR T-cell infusion and is largely treated with broad-spectrum antibiotics. However, confirmed bacterial documented infections after the first fever post–CAR T-cell infusion are very unusual. Because clinical parameters and biomarkers are not useful for identifying infectious fever, other methods should be assessed to ensure the proper use of antibiotics.

## Teaser

Confirmed bacterial infections are very unusual after the first episode of fever following CAR T-cell infusion. Clinical parameters and biomarkers are not useful for identifying infectious fever.

CD19 and B-cell maturation antigen (BCMA)–targeted chimeric antigen receptor (CAR) T-cell immunotherapies are effective treatments that have drastically improved outcomes in patients with refractory B-cell malignancies and multiple myeloma [1–3]. Following CAR T-cell infusion, it is prevalent for patients, often neutropenic patients, to experience episodes of fever commonly treated with broad-spectrum antibiotics. Consequently, high rates of *Clostridioides difficile* and multidrug-resistant infection have been described often in this population days and months after CAR T-cell therapy [4, 5]. However, the fever experienced after infusion can be predominantly caused by different pathogenic mechanisms, such as infection or inflammatory response related to CAR T-cell treatment [6–8].

Studies comprehensively catalog all infectious episodes following the injection of CAR T cells, with rates ranging from 33% to 53% depending on the follow-up period (1, 2, 3, and even 12 months) [9–14]. However, none specifically explore the first febrile episode that follows the infusion in the first weeks, during which the question of etiology between infection and cytokine release syndrome is primarily raised. Furthermore, there is a lack of current information regarding the clinical and biological features to distinguish between infection and inflammatory response. Therefore, the aim of this study was to identify documented infections associated with post–CAR T-cell infusion initial fever and to evaluate daily changes in vital signs, laboratory parameters, and the National Early Warning Score (NEWS), an objective scale for assessing clinical stability in hospitalized patients [15, 16], among hematologic patients with bacterial documented infection and in those without documented infection associated with fever following CAR T-cell infusion. Our goal was to identify parameters that could facilitate antibiotic de-escalation in this specific population in the first fever post–CAR T-cell therapy infusion.

## METHODS

### Study Design and Setting

This was a retrospective, observational cohort study conducted at the Hospital Clinic of Barcelona (Spain), a 700-bed university center with 30 beds allocated to patients with hematological malignancies. From 2017 onward, patients from our institution have received therapy with our own anti-CD19 CAR T-cell product, varnimcabtagene autoleucel (ARI-0001 cells) [17]; our own anti-BCMA product, cesnicabtagene autoleucel (ARI0002h) [18–21]; and commercial products. Both of the in-house products are locally manufactured at the Hospital Clinic of Barcelona and consist of autologous, second-generation (4-1BB and CD3z based) CAR T-cell products targeting CD19 (ARI-0001) or BCMA (ARI0002h). Both therapies are able to redirect the antitumor activity of autologous T cells to target and eliminate CD19-positive or BCMA-positive cells present both in B-cell malignancies (B-cell lymphomas and leukemias) and plasma cell malignancies (multiple myeloma, plasma cell leukemia, and amyloidosis), respectively. Both products are manufactured locally using the CliniMACS Prodigy bioreactor and are cryopreserved until their administration in a fractionated manner. According to the hospital protocol, antimicrobial prophylaxis consists of acyclovir 400 mg or valacyclovir 500 mg twice a day for seropositive individuals with either herpes simplex or varicella zoster virus. It further includes levofloxacin 500 mg daily and fluconazole 400 mg daily while the absolute neutrophil count is <500 cells/μL, and trimethoprim 160 mg/sulfamethoxazole 800 mg 3 times weekly, post–neutrophil recovery and until 3 months after CAR T-cell infusion. The serum immunoglobulin G (IgG) concentration is assessed before and approximately a month after CAR T-cell infusion; immunoglobulin (400 mg/kg, intravenous) reposition is recommended if the serum IgG concentration is <400 mg/day and the patient has been diagnosed with “recurrent infections.” In general, patients who experience postinfusion fever are neutropenic and are managed according to a protocol based on the recommended standard of care approach to febrile neutropenia.

### Selection of Participants

We included all consecutive adult patients with hematological malignancies who received CAR T-cell therapy between January 2020 and July 2022. Data were automatically retrieved from the patients’ electronic health records (EHRs) after a 9-step quality review pathway to assess data quality (Supplementary Figure).

We collected information regarding demographics, underlying malignancy and comorbidities, and type of CAR T-cell product used (ARI001 [varnimcabtagen autoleucel], axicabtagene ciloleucel, lisocabtagene maraleucel, tisagenlecleucel, or ARI0002h [cesnicabtagene autoleucel]). Clinical characteristics, laboratory data, and microbiological features were retrieved daily during hospitalization. For patients who had fever (temperature ≥38°C) in the 30 days following CAR T-cell infusion, we looked for vital signs, laboratory and microbiological data, treatment, and outcomes. NEWS [15, 16] was calculated at fever onset (day 0) and every day until day 3 to objective assess the clinical deterioration in our adult patients.

The management of febrile neutropenic patients in our institution is extremely protocolized with exhaustive exploration and systematic sampling of peripheral venous blood, from the central catheter, urine, respiratory viruses and pathogens, and any other infectious focus. Patients are then placed on empirical antibiotic therapy, taking into account their history of infection and colonization with multiresistant bacteria. Every morning, a team of infectious disease specialists reviews all the records of hematological patients presenting with fever and conducts very close monitoring, adjusting the antibiotic therapy based on the microbiological results and the clinical progression of the patient.

The Hospital Clinic Ethics Committee approved this study (number HCB/2022/0958) and waived the requirement for informed consent due to the retrospective study design and full anonymization of the data.

### Analysis

We reported descriptive statistics as median with interquartile range (IQR) for continuous variables and absolute numbers with percentages for categorical variables. For patients who had fever during the 30 days following CAR T-cell infusion, the values of vital signs and laboratory tests were retrieved during the first 3 days after fever onset (day 0) in patients without documented infection and in those with documented bacterial infection. Values at days 1, 2, and 3 were compared to day 0 in each group and between groups at fever onset (day 0) using the Mann-Whitney test. The threshold for statistical significance was defined as 2-tailed *P* < .05. Graphs were plotted and data analyzed with R software version 4.2.2 (R Foundation for Statistical Computing, Vienna, Austria).

## RESULTS

### General Characteristics

During the study period, 152 patients with hematological malignancies received CAR T-cell therapy. Their characteristics are reported in Table 1. A total of 87 (57.2%) patients had fever within 30 days following CAR T-cell infusion, with a median time from infusion to fever of 3 (IQR, 2–5) days. Patients with fever received antibiotics within 3 days of fever onset in 82 (94.3%) cases and tocilizumab in 51 of 87 (58.6%) cases. The antibiotics prescribed are listed in Table 2. The median time to tocilizumab prescription after infusion was 6 (IQR, 5–9) days.

**Table 1.** General Characteristics of Patients Who Received Chimeric Antigen Receptor T-Cell Therapy

Variable	No. (%)
No.	152
Sex (female)	85 (55.9)
Age, y, median (IQR)	55 (38–65)
Hematologic malignancy	
Non-Hodgkin lymphoma	61 (40.1)
ALL	59 (38.8)
Multiple myeloma	34 (22.4)
HSCT	92 (60.5)
Comorbidities	
Hypertension	25 (16.4)
Previous solid tumor	23 (15.1)
Chronic heart disease	22 (14.5)
Diabetes mellitus	9 (5.9)
Chronic pulmonary disease	7 (4.6)
Chronic liver disease	6 (3.9)
Chronic kidney disease	5 (3.3)
Solid organ transplant	1 (0.7)
CAR T-cell therapy	
Academic-ARI001	121 (79.6)
Axicabtagene ciloleucel	27 (17.8)
Lisocabtagene maraleucel	2 (1.3)
Tisagenlecleucel	2 (1.3)
Days between timepoints, median (IQR)	
Admission and infusion	1 (1–6)
Infusion and tocilizumab	6 (5–9)

Data are presented as No. (%) unless otherwise indicated.

Abbreviations: ALL, acute lymphoblastic leukemia; CAR, chimeric antigen receptor; HSCT, hematopoietic stem cell transplant; IQR, interquartile range.

**Table 2.** Prescribed Antibiotics Within 3 Days of Fever Onset

Antibiotic	No. (%)
No.	87
Meropenem	55 (63.2)
Piperacillin-tazobactam	24 (27.6)
Teicoplanin	20 (23.0)
Levofloxacin	20 (23.0)
Ertapenem	5 (5.7)
Amikacin	5 (5.7)
Vancomycin	4 (4.6)
Others	12 (13.8)

A total of 9 (10.3%) patients had a documented infection: 4 (4.6%) with bacteria, 4 with a virus, and 1 with both candidemia and viral infection (1.1%). Three patients tested positive for severe acute respiratory syndrome coronavirus 2. In 2 of these cases, CAR T-cell infusion was performed with prior knowledge of viral infection. Table 3 details documented infections.

**Table 3.** Documented Infections in Patients With Initial Fever Post–Chimeric Antigen Receptor T-Cell Infusion

Patient	Bacteria	Virus	Fungi
1	*Staphylococcus epidermidis* (BC and catheter)	…	…
2	…	Coronavirus (BAL)	…
3	…	SARS-CoV-2 (NPS)	…
4	…	SARS-CoV-2 (NPS)	*Candida parapsilosis* (BC)
5	…	RSV (NPS)	…
6	*Enterococcus faecium* (BC)	…	…
7	*Enterococcus faecalis* (BC)	…	…
8	*Staphylococcus haemolyticus* (BC)	…	…
9	…	SARS-CoV-2 and other coronavirus (NSP)	…

Abbreviations: BAL, bronchoalveolar lavage; BC, blood culture; NSP, nasopharyngeal swab; RSV, respiratory syncytial virus; SARS-CoV-2, severe acute respiratory syndrome coronavirus 2.

Table 4 details the vital signs, NEWS, and laboratory results for the 87 patients at fever onset (day 0) after CAR T-cell infusion and their evolution within the first 3 days in patients depending on whether they had a documented bacterial infection or no documented infection. At fever onset, there were no differences among those parameters between patients without infection and those with documented bacterial infection, except for NEWS, which was higher in patients without infection (5 [IQR, 4–6] vs 3 [IQR, 3–3]; *P* = .03). Tachycardia and hypotension were common in the first 3 days, both in patients with bacterial documented infection and in those without documented infection. In patients with bacterial documented infection, the median temperature on day 3 postinfusion and after appropriate treatment was 37°C (IQR, 36.7°C–37.6°C), whereas in patients without documented infection, the temperature tended to remain high (37.8°C [IQR, 37.1°C–38.4°C]). The NEWS did not show any significant clinical changes in any group. On day 3, there were no differences among those parameters between patients without documented infection and those with bacterial documented infection.

**Table 4.** Evolution of Vital Signs, National Early Warning Score, and Laboratory Results at Fever Onset and Each Day Until Day 3 in Patients Who Received Chimeric Antigen Receptor T-Cell Therapy and Whether They Had a Documented Bacterial Documented Infection or No Documented Infection

Variable	Documented Infection	Day 0	Day 1	Day 2	Day 3
Temperature, °C	No documentation	38.2 (38.1–38.5)	38.1 (37.6–38.6)	38.2 (37.6–38.8)	37.8 (37.1–38.4)
	Bacterial documentation	38.1 (38.0–38.2)	38.1 (37.6–38.6)	37.5 (37.2–37.8)	37.0 (36.7–37.6)
Heart rate, bpm	No documentation	106 (100–114)	106 (98–113)	105 (98–116)	103 (93–113)
	Bacterial documentation	96 (95–98)	97 (94–108)	87 (83–94)	98 (84–115)
SBP, mm Hg	No documentation	94 (86–103)	94 (86–103)	94 (86–104)	96 (85–104)
	Bacterial documentation	101 (101–101)	95 (94–96)	96 (95–102)	103 (96–107)
DBP, mm Hg	No documentation	58 (52–65)	57 (50–65)	57 (52–66)	57 (52–66)
	Bacterial documentation	57 (55–62)	54 (53–56)	56.5 (53–58)	61 (58–64)
Respiratory rate, cpm	No documentation	18 (17–20)	18 (16–20)	18 (16–20)	18 (16–20)
	Bacterial documentation	16 (16–17)	16 (16–17)	16 (16–18)	20 (18–20)
Oxygen saturation, %	No documentation	96 (95–98)	96 (95–98)	96 (95–97)	96 (95–97)
	Bacterial documentation	97 (97–97)	97 (97–98)	98 (98–98)	97 (96–97)
NEWS	No documentation	5 (4–6)	4 (3–5)	4 (3–6)	4 (3–6)
	Bacterial documentation	3 (3–3)	3 (3–4)	2 (2–3)	2 (1–4)
CRP, mg/dL	No documentation	2.08 (0.98–3.64)	4.36 (2.83–7.46)	5.59 (3.58–9.99)	5.27 (2.82–9.64)
	Bacterial documentation	2.37 (1.88–3.58)	7.23 (4.54–9.90)	6.36 (4.92–8.63)	10.68 (7.38–11.81)
Ferritin, ng/mL	No documentation	739 (282–1280)	721 (303–1556)	989 (385–1746)	1124 (503–2685)
	Bacterial documentation	1183 (935–1432)	1469 (1176–1763)	2078 (1585–2570)	… (…)
Lymphocytes, 10^9^/L	No documentation	0.10 (0–0.20)	0.10 (0–0.20)	0.10 (0–0.20)	0.10 (0–0.20)
	Bacterial documentation	0.80 (0.40–0.85)	0.40 (0.18–0.75)	0.25 (0.08–0.75)	0 (0–0.15)
Neutrophils, 10^9^/L	No documentation	0.40 (0.10–1.00)	0.20 (0.10–0.80)	0.30 (0.10–0.60)	0.20 (0.10–0.40)
	Bacterial documentation	0.40 (0.25–30)	1.65 (0.15–3.20)	1.20 (0.18–2.22)	0.10 (0.05–1.45)

Data are presented as median (interquartile range).

Abbreviations: CRP, C-reactive protein; DBP, diastolic blood pressure; NEWS, National Early Warning Score; SBP, systolic blood pressure.

## DISCUSSION

In this study, more than half of the patients treated with CAR T-cell experienced fever postinfusion. Of these, <5% had documented bacterial infections. Clinical parameters and biomarkers at fever onset were similar in patients with documented infection and in those without documented infections. Despite the low incidence of bacterial infection in the first fever episode post–CAR T-cell infusion, almost all of these patients received broad-spectrum antibiotic therapy. This discrepancy between the low number of proven bacterial infections and systematic use of broad-spectrum antibiotics in these patients raises major questions. Indeed, in addition to the risks of adverse events and drug interactions related to certain antibiotics, the main danger is promoting the emergence of resistance. Infection with *C difficile* represents a significant threat in this population, as it is the most common bacterial microorganism isolated in early infections following CAR T-cell infusion [4, 5] and has been reported to range from 12.5% to 30% [5, 22]. Moreover, a restrictive antibiotic treatment strategy has shown to protect microbiota in neutropenic allogeneic stem cell recipients with early fever due to cytokine release syndrome, without increasing the risk of infectious complications [22].

Our infection rate was lower than that reported in other studies [5, 9, 10]. However, it is important to note that in contrast to previous reports, we focused on the first fever episode following CAR T-cell infusion. The policy of antimicrobial prophylaxis protocols at our institution may also play a role. It is also interesting to note that there was a discrepancy between the spectrum of empirically initiated antibiotic therapies at the onset of fever, which primarily targeted gram-negative bacteria, and the type of bacteria identified, which were predominantly gram-positive. This finding is likely related to the fact that the first fever post-CAR T-cell infusion, when not inflammatory, was due to catheter sepsis. We did not find cases of endogenous bacteremia in this situation, as we are accustomed to seeing in other scenarios of patients with febrile neutropenia.

Identifying a pattern that could distinguish between the inflammatory response and infection in fever onset is the most important challenge for the antibiotic decision-making process. In our study, we observed no differences in fever onset between patients without documented infection and those with bacterial documented infection. Importantly, clinical parameters, such as temperature or heart rate, remained high during the first days after CAR T-cell infusion, even when no infection was documented. This finding is significant because these clinical parameters should not be used to decide whether antibiotics should be continued. Our study suggests that patients with fever following CAR T-cell infusion who do not have an infection do not show significant variations in NEWS. The NEWS is an objective scale created to identify patients at risk of deterioration [15, 16].

Further studies should assess whether a bundle that includes negative microbiological results and NEWS stability despite persistent fever and tachycardia is safe and useful for improving antibiotic stewardship in these patients.

Our study had several limitations. First, it was a single-center study that uses antibiotic prophylaxis in this population. Infection rates may differ in the absence of bacterial prophylaxis. Second, due to the retrospective design, infectious suspected clinically without microbiological documentation were not considered. The decision not to report this suspected clinical infection was based on 2 concepts: (1) the methodology used for the study (data directly retrieved from EHRs) is very strong in objective data and weak in subjective data; and (2) the utility of syndromic-based reporting (eg, pneumonia) is limited, especially in this population, by factors such as the challenge in differentiating inflammation from infection, and the subjectivity and/or inconsistent definitions of suspected infections used across studies [23].

Third, the study focused on the first episode of fever following CAR T-cell infusions during hospital stay and did not consider subsequent infectious episodes.

In conclusion, fever is a common symptom following CAR T-cell infusion and is largely treated with broad-spectrum antibiotics. However, confirmed bacterial infections are very unusual. Just as clinical parameters and biomarkers are not useful for identifying infectious fever, other parameters must be determined to ensure proper use of antibiotics. Bundles including clinical stability and ruling out of bacterial documented infections may help clinicians to stop antibiotics early to avoid emergence of resistance and infections due to *C difficile.*

## Supplementary Material

ofae398_Supplementary_Data

## Supplementary Data

Supplementary materials are available at *Open Forum Infectious Diseases* online. Consisting of data provided by the authors to benefit the reader, the posted materials are not copyedited and are the sole responsibility of the authors, so questions or comments should be addressed to the corresponding author.

## Notes

***Acknowledgments.*** The authors thank Anthony Armenta for providing medical language editing assistance for the manuscript at hand. O. P. received postdoctoral fellow financial support from la Ligue Nationale contre le Cancer (convention number: AAPMRC 2022/OP) and la Direction de l’Assistance Publique–Hôpitaux de Paris.

***Author contributions.*** Conception and design: O. P., N. G.-P., and C. G.-V. Provision of study materials of patients: All authors. Collection and assembly of data: O. P., N. G.-P., M. C., A. S., and C. G.-V. Data analysis and interpretation: O. P., N. G-P., and C. G.-V. Manuscript writing: O. P., N. G.-P., and C. G.-V. Final approval of manuscript: All authors.

***Ethical approval.*** The Hospital Clinic Ethics Committee approved this study (number HCB/2022/0958); informed consent was waived because of the retrospective study design and full anonymization of the data.

***Patient consent.*** This study does not include factors necessitating patient consent.

***Disclaimer.*** The funders had neither a specific role in study design or data collection, nor in the writing of the paper or decision to submit for publication.

***Financial support.*** This work was supported by the European Regional Development Fund. C. G.-V. (FIS PI21/01640 and ICI21/00103) and P. P.-A. (JR20/00012 and PI21/00498) received research grants from the Ministerio de Sanidad y Consumo, Instituto de Salud Carlos III and co-funded by the European Union under the Next Generation EU funds, which finance the actions of the Recovery and Resilience Facility (RRF/RTRP). This work has been supported in part by grants from the Instituto de Salud Carlos III (co-funded by the European Union), Spanish Ministry of Health (ICI19/00025 and PI22/00647), RICORS-TERAV network (RD21/0017/0009 and RD21/0017/0019), Red de Terapia Celular TerCel (RD16/0011/0005), Fondo Europeo de Desarrollo Regional (FEDER), Agencia de Gestión de Ayudas Universitarias y de Investigación (AGAUR), Generalitat de Catalunya (2017SGR00792 and SGR 01324 Q5856414G), La Caixa Foundation (CP042702/LCF/PR/GN18/50310007), and Asociación Española Contra el Cancer (LABAE21971FERN). T. F. A. has received a pre-doctoral grant supported by the Ministerio de Sanidad y Consumo, Instituto de Salud Carlos III [RH RH042953]. The present study is co-financed by the Instituto de Salud Carlos III, with file code CM23/00277, under the Resolution of the Directorate of the Instituto de Salud Carlos III, O. A., M. P. of December 13, 2023, granting the Rio Hortega Contracts, and co-financed by the European Union and by the European Social Fund Plus (ESF+).
