# Supplementary figures and images for: Chimeric Antigen Receptor T-Cell Postinfusion Fever: Infection Profile, Clinical Parameters, and Biomarkers Trends to Assist Antibiotic Stewardship
Source: Open Forum Infect Dis. 2024 Jul 11;11(7):ofae398. doi: 10.1093/ofid/ofae398 (PMC11273324; doi:10.1093/ofid/ofae398)

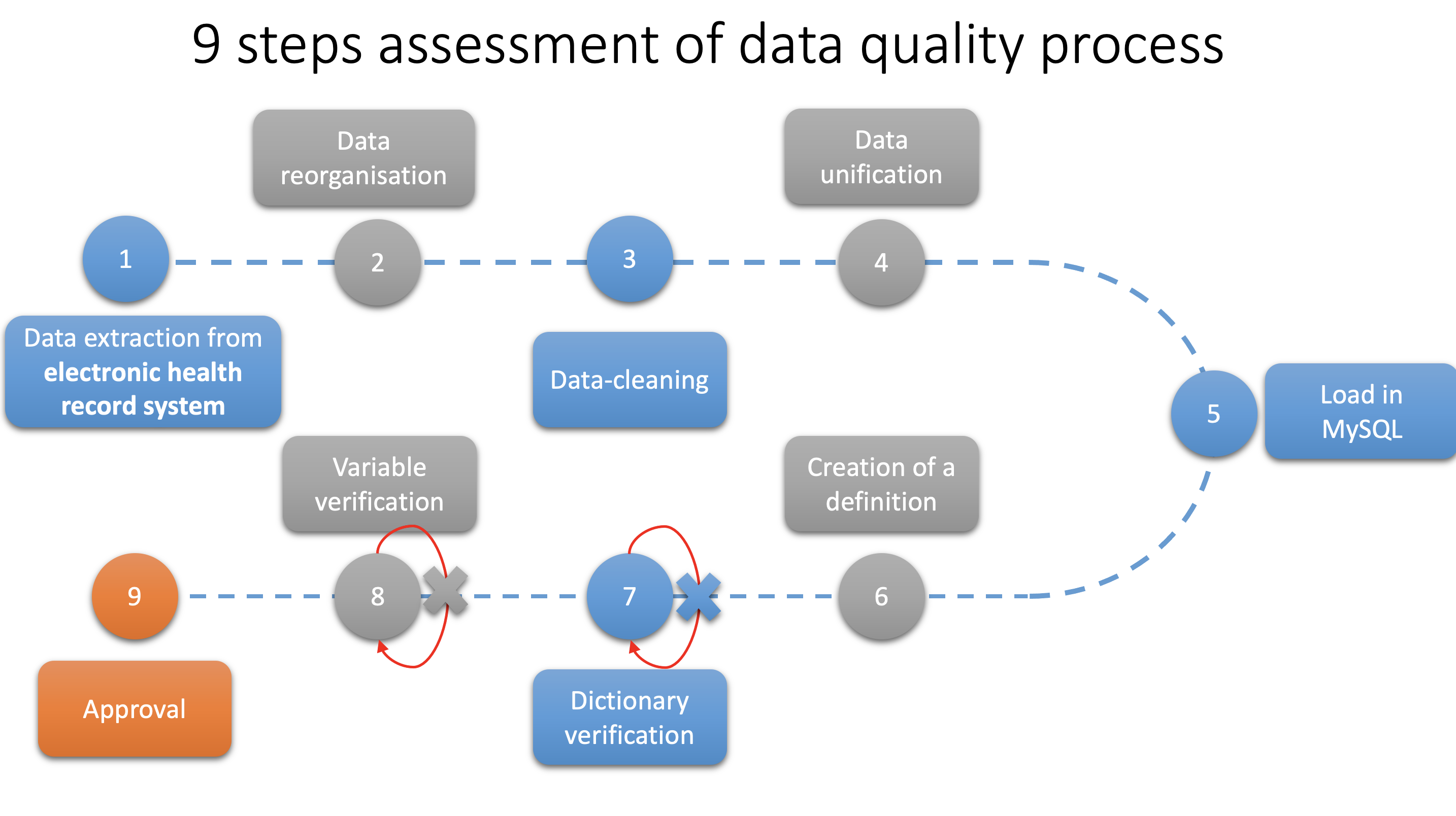

Supplement: ofae398_Supplementary_Data [file ofae398_supplementary_data.zip › Supplemental Digital Content 1 (Figure).png]
